# Biological Water Quality Indices Performance Based on Aquatic Insects in Recreational Rivers

**DOI:** 10.21315/tlsr2021.32.1.6

**Published:** 2021-03-31

**Authors:** Ahmad Hadri Jumaat, Suhaila Abdul Hamid

**Affiliations:** School of Biological Sciences, Universiti Sains Malaysia, 11800 USM Pulau Pinang, Malaysia

**Keywords:** Water Quality, Biological Indicator, Pollution, Recreational River, Kualiti Air, Penunjuk Biologi, Pencemaran, Kawasan Rekreasi Sungai

## Abstract

Abundance and distribution of aquatic insects respecting to several water chemical parameters from six rivers were studied in order to determine the performance of biological index in monitoring the water quality. A total of 960 individuals of aquatic insects from nine orders were recorded using kick and drag sampling techniques. Lubok Semilang had the greatest number of aquatic insects with 250 individuals, followed by Telaga Tujuh (181 individuals) and Sungai Durian Perangin (171 individuals). EPT (Ephemeroptera, Plecoptera and Trichoptera) order were the most dominant order recorded in all six rivers. Lata Kekabu had more diverse and richer aquatic insect assemblages based on ecological indices compared to the other five rivers. In order to evaluate the water quality of recreational rivers in Malaysia, Family Biotic Index (FBI), Malaysian Family Biotic Index (MFBI) and Biological Monitoring Working Party (BMWP) were used and compared with Water Quality Index (WQI) to determine the water quality at the study areas. Results demonstrated that the biotic indices were more sensitive towards changes in water parameters than the WQI. Among all the biological indices, MFBI was the most suitable index to be adopted in Malaysian river water assessment as it is more reliable in assessing the status of water quality.

## Abstract (ms)

Kelimpahan dan taburan serangga akuatik serta parameter kimia air di enam kawasan sungai telah dikaji untuk melihat prestasi indeks biologi dalam pemantauan tahap kualiti air sungai. Sejumlah 960 individu daripada sembilan order serangga akuatik telah direkod dengan menggunakan teknik persampelan secara *kick and drag*. Lubok Semilang telah mencatatkan bilangan serangga akuatik yang tertinggi dengan 250 individu diikuti oleh Telaga Tujuh (181 individu) dan Sungai Durian Perangin (171 individu). Order Ephemeroptera, Plecoptera and Trichoptera (EPT) didapati mendominasi kesemua sungai. Lata Kekabu mempunyai taburan dan kekayaan serangga akuatik yang tinggi berdasarkan semua indeks ekologi jika dibandingkan dengan lima sungai yang lain. Untuk menilai kualiti air di kawasan rekreasi di Malaysia, penggunaan indeks biologi seperti Family Biotic Index (FBI), Malaysia Family Biotic Index (MFBI) dan Biological Monitoring Working Party (BMWP) telah dibanding dan diguna bersama dengan Water Quality Index (WQI) untuk menentukan kualiti air di semua kawasan kajian. Keputusan menunjukkan bahawa indeks biotik merupakan indeks yang lebih sensitif terhadap perubahan parameter air berbanding WQI. Di antara semua indeks biologi yang dikaji di Malaysia, MFBI merupakan indeks yang paling sesuai digunakan di Malaysia kerana lebih efisien dalam menilai status kualiti air sungai.

**Highlights**

- Lubok Semilang had the greatest number of aquatic insects with 250 individuals, followed by Telaga Tujuh (181 individuals) and Sungai Durian Perangin (171 individuals).
- Family Biotic Index (FBI), Malaysian Family Biotic Index (MFBI) and Biological Monitoring Working Party (BMWP) were used and compared with Water Quality Index (WQI) to determine the water quality at the study areas.
- Biotic indices were more sensitive towards changes in water parameters than the WQI.

## INTRODUCTION

Land use activities have affected the condition of aquatic ecosystems worldwide including Malaysian that cause river physical modification, habitat loss, water pollution and flora and fauna overexploitation. According to Hepp *et al.* (2013), most water bodies such as rivers has consequently been subjected to increasing the quantity of pollutants present in a river, affecting greatly their quality and health status. This definitely alters the physicochemical properties of water. Variations in these water properties greatly influence the structure and distribution of aquatic insects in the water (Hepp *et al.* 2013). There are many methods and techniques developed by the researchers to analyse the impairments of water quality in aquatic ecosystem. For example, a study done by Aweng *et al.* (2011) and Zarei and Bilondi (2013) indicated that analyses of both physical parameters (turbidity, sedimentation, siltation, flow patterns, water temperature and riparian cover) and chemical parameters [dissolved oxygen (DO), biochemical oxygen demand (BOD), pH, alkalinity, metals and organic compounds] have been investigated further to assess the quality of water.

Above and beyond, biological monitoring act as the main principal of indicator towards the two major condition which is past conditions as well as current conditions (Suhaila *et al.* 2014). Oliveira and Cortes (2006) stated that, the supplemental integration of biological parameters to physical-chemical assessments has proven to be a more complete method to fully assess pollutant effects in aquatic ecosystems most particularly in river and streams area. According to Engel and Voshell (2002), biological monitoring is defined as scientifically and economically valid approach evaluation of the condition of a water body. The evaluation process is specifically using biological monitors and other direct measurements of the biota in surface waters for indicator and monitoring program to assess the water quality of rivers or streams (Kopciuh *et al.* 2004). Based on the definition above, there are many advantages of using the biological assessment to evaluate the water quality of rivers. Biological assessment is more reliable in evaluating the presence and impact of pollutants in water. Benthic macroinvertebrates and aquatic insects have been used in numerous biological monitoring indicating their usefulness as bioindicators and the continuous advantages they offer in evaluating the presence and extent of environmental pollutants (Xu *et al.* 2014).

Among the benthic macroinvertebrates, aquatic insect is one of the most common group of organisms that can be used to determine the river condition (Lavoie & Campeau, 2010). According to Xu *et al.* (2014) aquatic insects have been chosen as the useful indicator to assess the water quality of the rivers because they represent a diverse and various group of sedentary organisms that react strongly to environmental changes. For example, some aquatic insects are highly responded to specific changes in water conditions and have become indicators of river health condition (Xu *et al.* 2014) compared to fish and other macroinvertebrates. This can be seen when aquatic insects provide a more accurate understanding of the changing freshwater ecosystem than chemical and physical monitoring. Responsive behaviour of aquatic insect towards the pollution or contaminations gives an early warning to any possible harm of the water resources. To date, very few studies on the community of aquatic insects in recreational river as biological indicator has been carried out as most of the studies give focused on chemical pollution in contaminated rivers (Al-Shami *et al.* 2013; Al-Shami *et al.* 2011). Others preferred to concentrate on the diversity and abundance of benthic fauna (Suhaila *et al.* 2011; Che Salmah *et al.* 2012).

Based on the research by Leunda *et al.* (2009), although there were local researches that adopting biological indices which were developed in temperate countries for water quality assessment in Malaysia but it would be less accurate and thus requires some modification (Hilsenhoff 1988) as being practiced in Thailand (Mustow 2009) and Vietnam (Hoang 2009). Meanwhile in 2016, Wan Mohd Hafezul has developed a Malaysian Family Biotic Index (MFBI) based on Malaysia aquatic insects. Thus, this study was carried out to determine the diversity, abundance and composition of aquatic insect at selected recreational rivers and to evaluate the performance of MFBI with other establish biological indices.

## MATERIALS AND METHODs

### Description of Study Area

This study was carried out at six selected rivers: Lata Kekabu and Sungai Gelok in Perak, and Sungai Sedim, Sungai Durian Perangin, Lubok Semilang and Telaga Tujuh in Kedah. Lata Kekabu and Sungai Gelok are frequently visited by the local and located 10 km from Lenggong, Perak. Meanwhile, Sungai Sedim is located within the Gunung Inas Forest Reserve. Sedim Recreation Park is the only location for white water rafting located in the north-west states and been classified as lowland dipterocarp forest by Forestry Department of Peninsular Malaysia. Durian Perangin Waterfall is located on the northern slope of Gunung Raya, the highest mountain in Langkawi. This river was surrounded by dipterocarp trees along the river margins and has partly shaded canopy cover. Lubok Semilang is an open shaded area and this river substrate consisted of 70% cobble, 15% gravel, 10% boulder and 5% sand. Nevertheless, there were not many visitors at this river as compared to other famous recreational river like Durian Perangin Waterfall and Telaga Tujuh Waterfall that provide many water sports activities. Telaga Tujuh Waterfall is an open area and was surrounded by many type of plant like unique lime plants and sintuk, a climbing type of foliage, which grow abundantly.

### Sampling and Identification of Aquatic Insects

Samples of aquatic insect communities were carried out using kick sampling techniques which is a modified technique of Merritt *et al.* (2008). Ten samples were collected during the sampling occasional at each sampling site by random samples across approximately 100 m stretch in each river using D-frame aquatic net. The content of each sample was transferred into properly labelled plastic bags filled with a small amount of river water, tied by rubber band and was brought to the laboratory for further laboratory work. In the laboratory, each bag was washed and sorted in a tray. Sorted aquatic insects were preserved in bottles that contained 75% ethanol (ETOH). Later, the aquatic insects were identified to the lowest taxa using reference keys from Yule and Yong (2004), Morse (2004), Webb and McCafferty (2008) under a stereo microscope (LEICA EZ4, Leica Microsystems (SEA) Pte Ltd., Singapore).

### Physicochemical Parameter Measurements

DO, pH, total suspended solids (TSS) were measured *in situ* by using MPS YSI 556/550A (Yellow Spring Instrument, OH, US) which is a multi-parameter handheld meter. Water samples were collected concurrently with the aquatic insects sampling. The polyethylene bottle was rinsed with river water first before the actual sample was taken. Chemical oxygen demand (COD), BOD, ammonia-nitrogen (NH_3_-N) and TSS were analysed in the laboratory using a standard kit of HACH DR/900 Calorimeter (HACH Company, Loveland, USA).

### Data Analysis

Pearson Correlation Analysis was used to measure the association between aquatic insect abundance and physico-chemical parameters using SPSS version 23. Biological Monitoring Working Party (BMWP), Average Score Per Taxon (ASPT), FBI and MFBI were calculated to classify the river water quality (Table 1). Malaysian tolerance value and formula for MFBI calculation was followed from Wan Mohd Hafezul (2016).

## RESULTs

A total of 960 individuals from 32 families of aquatic insects from 9 order (Coleoptera, Diptera, Ephemeroptera, Hemiptera, Odonata, Plecoptera, Trichoptera, Lepidoptera and Megaloptera) were collected from all rivers (Table 2). Most of the aquatic insects were collected from Lubok Semilang (26%) followed by Telaga Tujuh (18.9%), Sungai Durian Perangin (17.8%) and Lata Kekabu (17%). Meanwhile, Sungai Gelok had a lower number of aquatic insects collected which less than 15%, followed by Sungai Sedim which only had 7% with 67 number of individuals collected. Kruskal Wallis test showed that the abundance of aquatic insects among the six selected rivers (*χ*^2^ = 9.17, *P* = 0.10) are not statistically significant. Stoneflies (Plecoptera), mayflies (Ephemeroptera) and caddisflies (Trichoptera) were the most dominant order in all selected rivers. In area of Perak, Lata Kekabu and Sungai Gelok showed a relatively high abundance of Perlidae (Plecoptera) with 76 individuals were collected, followed by Simuliidae (Diptera) with 45 individuals and Hydropsychidae (Trichoptera) with a total of 29 individuals (Table 3).

In Kedah, Lubok Semilang indicated the greatest abundance of Baetidae (Ephemeroptera) with 57 individuals and 50 individuals from the family of Hydropsychidae. On the other hand, Hydropsychidae was the most dominant family in Sungai Durian Perangin with 36 individuals. In contrast, Telaga Tujuh had the greatest number of Simuliidae (Diptera) with 44 individuals compared to Baetidae (37 individuals) and Perlidae (30 individuals). Based on the Table 4, pH and Ammonia Nitrogen (NH_3_-N) had a negative correlation with aquatic insect abundance in all six selected rivers with (*r* = −0.122, *P* = 0.073) and (*r* = −0.061, *P* = 0.373), respectively. Throughout the sampling period at all the six rivers, there was a significant relationship between aquatic insect abundance and several water parameters (DO and TSS). Telaga Tujuh had the highest concentration value of DO with 8.79 mg/L while Sungai Sedim had the lowest concentration compared to other rivers with only 7.23 mg/L.

The abundance of aquatic insects at all the six rivers had a considerable significant positive correlation with the concentration of DO (*r* = 0.175, *P* = 0.007). The highest value of TSS was recorded at Sungai Sedim with 32.37 mg/L, while the lowest value of TSS was 1.00 mg/L in Telaga Tujuh. There was a significant correlation between the TSS and abundance of aquatic insects (*r* = −0.140, *P* = 0.040). This indicate that the abundance of aquatic insects decreased as the TSS (dry weight of suspended particles that are not dissolved) in the rivers increased.

Furthermore, Sungai Gelok and Sungai Sedim were classified into Class II (Table 4) based on the Water Quality Index (WQI). Class II indicates that the water is still good and suitable for recreational use with body contact, but conventional treatment is required for livestock drinking. Lata Kekabu, Sungai Durian Perangin, Lubok Semilang and Telaga Tujuh had the high number of WQI value (Class I) which indicates that these rivers have better water quality compared to Sungai Gelok and Sungai Sedim.

In order to categorise the health of the streams based on aquatic insects composition and abundance, the FBI, MFBI and Biological Monitoring Work Party (BMWP) were calculated (Table 5). This index score equals the sum tolerance scores of all macroinvertebrate families with higher BMWP score is considered to reflect better water quality. For the BMWP index score, each aquatic insect family is calculated according to their sensitivity towards the organic pollution. Tolerance values are assigned from 1 (the most tolerant taxa) to 10 (the most sensitive taxa). Based on the value of BMWP, Lata Kekabu (165) and Sungai Gelok (134) shows good water quality while Sungai Sedim was classified as the moderately good according to the BMWP index. Scores from Sungai Durian Perangin (81), Lubok Semilang (80) and Tujuh Telaga (89) shows moderately good water quality according to the BMWP index.

Biotic Index (BI) was subsequently modified to the family-level with tolerance value ranging from 0 (very intolerant) to 10 (highly tolerant) based on their tolerance to organic pollution creating the FBI (Mandaville 2002). Based on the calculated value of FBI, Lata Kekabu (3.49), Sungai Sedim (2.33) and Sungai Durian Perangin (3.26) were classified as having very good water quality and organic pollution is unlikely. Meanwhile, Sungai Gelok (3.88) was classified under good water quality, even though there might be slight organic pollution.

## DISCUSSION

Those six selected rivers show different pattern of aquatic insect’s assemblages. Plecoptera (31%) was found dominating most of the river studied, followed by the Trichoptera (20%) and Ephemeroptera (16%). Meanwhile, the most dominant order was Plecoptera (24%) and Diptera (24%) found in Sungai Gelok and the second most dominant was Trichoptera (19%). In addition, family of ephemeropterans dominates the aquatic insect communities in Telaga Tujuh (39%) and Lubok Semilang (37%). In Telaga Tujuh, there are many dipteran families present with 181 individuals and Simuliidae is the most dominant family that was predominated in Telaga Tujuh (88%).

Members of Ephemeroptera, Plecoptera and Trichoptera (EPT) are considered to be sensitive towards the environmental stress, thus, their dominance in all six rivers signifies a relatively clean environment (Suhaila *et al.* 2014; 2017; Wan Mohd Hafezul 2016). EPT are very much intolerable to any presence of pollutants in the water bodies and thus EPT are crucial biological indicators in determining water quality of the river.

DO is the most important parameter in classifying a river’s class in WQI. Based on the coefficient of DO, it influences the water quality index by 22%. When the value of DO is lower, WQI tends to have lower value and if the value of DO is higher, it is vice versa. However, our study found that the high richness of EPT families (such as Baetidae and Hydropsychidae) of the river contributed to the high scores of all BMWP indices, thus classified the rivers into having clean water. In contrast, the presence of tolerant taxa such as chironomids in the river led to poor water quality classification by the BMWP indices. In this regard, it is safe to conclude that the BMWP displayed higher sensitivity towards organic pollutants in the river compared to the WQI.

According to Allan (2004), land use disturbance does impacts local habitat, features and diversity in the rivers. Aquatic insects react differently towards the several changes in water quality parameter (Batty 2005). Previous study by Rife and Moody (2004) showed EPT orders are sensitive to water quality changes with respects to their low levels of adaptive mechanisms. Based on the findings, it could be simplified that the water parameters in Lata Kekabu, Sungai Gelok, Sungai Sedim, Sungai Durian Perangin, Lubok Semilang and Telaga Tujuh are in tolerable limits since EPT are the dominant orders found.

According to Semenchenko and Moroz (2005), the water quality classification and wide application of BMWP for river water assessment is related to its simplicity and convenience for water evaluation. Unlike the FBI, the BMWPs considered all macroinvertebrate groups that contributed to the condition of the monitored rivers (Mustow 2009). For example, this study found that the high richness of EPT families (such as Perlidae and Baetidae) and dipteran Simuliidae in Lubok Semilang contributed to the high scores of BMWP, thus classified the river as clean water quality. In contrast, the presence of tolerant taxa such as chironomids and Tipulidae in Sungai Durian Perangin led to moderately good water quality classification by the BMWP index. This is because, the BMWP was calculated based on the tolerance score of each family of macroinvertebrate that were collected not their abundance.

In this study, the performances of three biotic indices were compared to biologically assess the water quality with tendencies of some indices to be more suitable for use in certain river conditions. The FBI, MFBI and BMWP are based on the sensitivity of key groups to pollution and on the number of component groups in a sample. Since many years ago, a lot of biological indices have been developed and used in many countries. Nevertheless, the development and performance of MFBI in this study is a step forward in the study of bioindicator and biological water assessment in Malaysia. The MFBI formula are specifically generate value to indicate definite condition of the rivers undergoing assessment with variant in terms of assemblages of the aquatic organisms in Malaysia. The MFBI are considering the abundance of taxa in the estimation of the index score that increase the accuracy of the river condition.

Therefore, five classes of water quality were suggested as for MFBI (Wan Mohd Hafezul 2016). These five classes are considered optimum and efficient in evaluating the water quality of the rivers. In this study, all the five rivers had been classified into the second class (Class II) that ranged between 4.5 to 5.9, referred to good water quality. Most of rivers in this range, recorded the composition of intolerant taxa such as Baetidae and Perlidae. Thus, rivers fell within range values were having good water quality.

In this study, the newly developed index which is MFBI (Wan Mohd Hafezul 2016) that used locally located taxa was assessed for their validity in order to confirm their accuracy and reliability for river health bioassessment. Changes in biotic index along the river should be consistent with changes in the water chemistry. It was found that the changes in MFBI scores were driven by oxygen content, BOD and nitrogen in the water (Wan Mohd Hafezul 2016). Consequently, the low oxygen contents contributed to the decrease of MFBI scores that indicated river impairment. For example, Telaga Tujuh and Lubok Semilang that recorded high DO (8.79 mg/L and 8.77 mg/L, respectively) had very high scores of the MFBI consequently fell into Class II (good water quality).

Inconsistence of the water categorisations among rivers by various indices were observed. Some rivers were classified into having “good” water quality by the MFBI and comparable to WQI (Class I) but “moderately good” water quality by the BMWP. Lubok Semilang was a good example for this case. This situation was presumably prompted by the presence of intolerant taxa such as Baetidae (tolerance value = 6) with high abundance. In addition, Lata Kekabu was classified as very good water quality by the BMWP, but the MFBI classified the rivers as good water quality. For this river, the MFBI classification was more appropriate because this river was surrounded by the residential area (chalet area) and recreational activities and its impairment was expected due to anthropogenic discharges. Therefore, the MFBI classified this river close to its actual condition. For instance, the MFBI and BMWP indices classified the river of Sungai Gelok into Class II (good water quality). Considering the differences and similarities of water classification by MFBI and BMWP index, the potential of the MFBI was comparable to the BMWP indices and the WQI, which indicated that the use of this MFBI in Malaysian rivers is reliable for water quality evaluation.

## CONCLUSION

Through this study, Lata Kekabu had the high abundance and diversity of aquatic insect collected compared to other rivers. Lata Kekabu had lesser recreational activity at the surroundings area which lead to the low level of chemical contamination in the water. EPT were the dominant order collected in all rivers. Based on the calculated WQI, Lata Kekabu, Sungai Durian Perangin, Lubok Semilang and Telaga Tujuh was classsified into Class I as having a very good water quality. Meanwhile, MFBI classified all rivers into Class II which is a good water quality. MFBI is more reliable to evaluate the water quality as the tolerance value for the taxa is derived from Malaysian specimens and are based on abundances of the taxa.

## Figures and Tables

**Table 1:** The MFBI classifications of the water quality.

Class	Index range	Category
1	> 5.9	Very good
2	4.5–5.8	Good
3	3.8–4.4	Moderately polluted
4	2.7–3.7	Polluted
5	< 2.7	Poor

*Source*: Wan Mohd Hafezul (2016)

**Table 2:** Abundance of aquatic insect collected from the selected rivers in Perak and Kedah, Malaysia.

Areas of sampling	Number of individuals	Percentage
Lata Kekabu	166	17.3
Sungai Gelok	125	13.0
Sungai Sedim	67	7.0
Sungai Durian Perangin	171	17.8
Lubok Semilang	250	26.0
Telaga Tujuh	181	18.9

Total	960	100.0

**Table 3:** Composition and abundance (mean ± SE) of aquatic insect collected from Lata Kekabu, Sungai Gelok, Perak and Sungai Sedim, Durian Perangin, Lubok Semilang and Telaga Tujuh, Kedah.

Order	Family	Genus	Lata Kekabu	Sungai Gelok	Sungai Sedim	Durian Perangin	Lubok Semilang	Telaga Tujuh
Ephemeroptera	Baetidae	*Baetis*	2.00 ± 0.0	1.00 ± 0.6	2.67 ± 0.7	4.67 ± 1.2	10.00 ± 1.2	7.67 ± 2.3
		*Platybaetis*	3.33 ± 0.7	1.33 ± 0.3	2.33 ± 0.3	3.00 ± 0.6	9.00 ± 0.6	4.67 ± 1.7
	Caenidae	*Caenis*	2.33 ± 0.7	-	-	0.67 ± 0.3	6.33 ± 1.2	3.33 ± 1.2
	Ephemeridae	*Ephemera*	0.66 ± 0.3	-	-	-	-	0.67 ± 0.3
	Heptageniidae	*Thalerosphyrus*	0.33 ± 0.3	0.67 ± 0.7	-	3.33 ± 0.9	2.33 ± 1.2	4.00 ± 1.5
	Isonychidae	*Isonychia*	-	1.00 ± 0.6	-	1.33 ± 0.9	1.67 ± 0.6	1.00 ± 0.6
	Trichorytidae	*Trichorythus*	-	-	-	-	1.33 ± 0.7	2.33 ± 0.9

Plecoptera	Perlidae	*Etrocorema*	5.00 ± 0.6	3.67 ± 0.9	2.33 ± 1.2	2.33 ± 1.3	4.33 ± 1.7	-
		*Neoperla*	8.00 ± 3.0	3.00 ± 0.6	2.67 ± 0.3	6.67 ± 2.2	5.00 ± 0.6	1.33 ± 0.7
		*Kamimuria*	3.33 ± 0.3	2.33 ± 1.5	1.33 ± 0.3	2.67 ± 0.3	0.67 ± 0.3	0.00 ± 0.0
	Peltoperlidae	*Cryptoperla*	1.00 ± 0.6	1.00 ± 0.6	-	3.67 ± 1.9	1.00 ± 0.6	-

Trichoptera	Hydropsychidae	*Hydropsyche*	3.33 ± 0.7	2.67 ± 1.3	1.00 ± 0.6	5.00 ± 1.5	0.67 ± 0.3	5.00 ± 1.0
		*Cheumatopsyche*	1.67 ± 0.7	1.67 ± 0.9	-	5.00 ± 1.2	7.00 ± 2.0	3.00 ± 0.6
		*Macrostermum*	-	0.33 ± 0.3	-	2.00 ± 0.6	3.67 ± 0.3	2.00 ± 1.5
	Polycentropodidae	*Polycentropus*	3.67 ± 1.5	2.67 ± 0.3	1.00 ± 0.6	1.67 ± 0.9	1.33 ± 0.7	1.33 ± 0.7
	Philopotamidae	*Chimarra spp.*	1.67 ± 0.7	0.67 ± 0.7	-	0.67 ± 0.3	-	-
	Stenopsychidae	*Stenopsyche*	0.67 ± 0.3	-	3.33 ± 1.3	1.00 ± 0.6	2.00 ± 0.9	0.67 ± 0.3
	Leptoceridae	*Triaenodes*	0.33 ± 0.3	-	-	-	-	-
	Lepidostomatidae	*Lepidostoma*	-	-	-	-	0.67 ± 0.2	-

Odonata	Corduliidae	*Onychotemis*	1.00 ± 0.6	0.33 ± 0.3	0.67 ± 0.3	0.33 ± 0.3	-	2.33 ± 1.5
	Euphaeidae	*Euphaea*	0.33 ± 0.3	1.00 ± 0.6	0.33 ± 0.3	-	-	-
	Gomphidae	*Nepogomphus*	1.00 ± 1.0	0.33 ± 0.3	-	-	-	1.00 ± 0.6
	Libellulidae	*Zygonyx*	1.33 ± 0.7	0.33 ± 0.3	0.67 ± 0.3	-	-	0.67 ± 0.7
	Platysticidae	*Platysticta*	-	-	0.33 ± 0.3	-	-	-

Coleoptera	Eulichadidae	*Eulichas*	1.00 ± 1.0	0.67 ± 0.3	3.33 ± 1.3	0.33 ± 0.3	0.33 ± 0.3	1.67 ± 0.9
	Elmidae	*Potamophilus*	1.00 ± 0.6	-	-	-	0.33 ± 0.3	-
	Hydrophillidae	*Laccobius*	2.00 ± 0.6	2.00 ± 1.2	-	0.67 ± 0.7	1.00 ± 0.6	-

Diptera	Simulidae	*Simulium*	7.00 ±1.7	8.00 ± 0.6	-	4.67 ± 1.9	14.00 ± 4.4	14.67 ± 4.6
	Tipulidae	*Hexatoma*	1.00 ± 0.6	2.00 ± 0.6	-	4.33 ± 1.2	0.67 ± 0.7	1.00 ± 0.6
	Chironomidae	*Chironomus*	-	-	-	2.33 ± 1.2	0.33 ± 0.3	1.33 ± 0.7

Hemiptera	Gerridae	*Metrocorsis*	-	-	0.33 ± 0.0	-	-	-
		*Rhagadotarsus*	-	0.33 ± 0.3	-	-	-	-
	Veliidae	*Rhagovelia*	-	4.67 ± 1.2	-	-	-	-
	Naucoridae	*Naucoris*	0.33 ± 0.3	-	-	-	-	-
	Apheloceridae	*Aphelocherius*	0.67 ± 0.3	-	-	-	-	-

Lepidoptera	Pyralidae	*Parapoynx*	1.00 ± 1.0	-	-	-	-	-

Megaloptera	Corydalidae	*Neochauliodes*	0.33 ± 0.3	-	-	-	-	0.67 ± 0.7

**Table 4:** Summary of water physico-chemical parameters (mean ± standard error) for Lata Kekabu, Sungai Gelok, Durian Perangin, Lubok Semilang, Telaga Tujuh and Sungai Sedim.

Parameter	Unit	River					

		Lata Kekabu	Sungai Gelok	Sungai Sedim	Durian Perangin	Lubok Semilang	Telaga Tujuh
Dissolved Oxygen (DO)	mg/L	8.67 ± 0.01	8.30 ± 0.00	7.23 ± 0.99	8.48 ± 0.01	8.77 ± 0.00	8.79 ± 0.04
Biochemcial Oxygen Demand (BOD)	mg/L	0.96 ± 0.07	1.17 ± 0.03	1.24 ± 0.00	0.26 ± 0.99	1.40 ± 0.03	0.29 ± 0.02
Chemical Oxygen Demand (COD)	mg/L	1.33 ± 0.88	1.00 ± 0.58	2.33 ± 2.33	8.00 ± 0.67	8.33 ± 0.55	8.10 ± 0.78
Ammoniacal Nitrogen (AN)	mg/L	0.02 ± 0.01	0.08 ± 0.01	0.01 ± 0.01	0.01 ± 0.00	0.01 ± 0.01	0.02 ± 0.02
Total Suspended Solids (TSS)	mg/L	23.10 ± 0.12	24.20 ± 0.12	32.37 ± 0.70	1.50 ± 0.00	4.50 ± 0.00	1.00 ± 0.00
Temperature	°C	23.50 ± 0.00	25.02 ± 0.00	23.20 ± 0.00	25.30 ± 0.01	25.00 ± 0.00	25.50 ± 0.02
Conductivity	S/m	23.00 ± 0.01	35.60 ± 0.02	13.03 ± 0.01	20.00 ± 0.33	25.00 ± 0.21	28.00 ± 0.26
pH	-	7.10 ± 0.05	7.50 ± 0.13	7.63 ± 0.15	6.72 ± 0.05	5.91 ± 0.04	8.23 ± 0.02
WQI	-	93.03	89.83	89.51	96.95	97.70	95.52
Class	-	I	II	II	I	I	I

**Table 5:** The classification of the rivers based on water quality indices (FBI, MFBI, BMWP and ASPT).

Biological Indices/Class	Lata Kekabu	Sungai Gelok	Sungai Sedim	Durian Perangin	Lubok Semilang	Telaga Tujuh
Family Biotic Indices (FBI)	3.49	3.88	2.33	3.26	4.27	4.27
Class	Very good	Good	Very good	Very good	Moderately good	Moderately good
Malaysia Family Biotic Indices (MFBI)	5.85	5.65	5.60	5.48	5.43	5.34
Class	Good	Good	Good	Good	Good	Good
Biological Monitoring Working Party (BMWP)	165	134	71	81	80	89
Class	Very good	Good	Moderately good	Moderately good	Moderately good	Moderately good
Average Score Per Taxon (ASPT)	6.71	6.92	7.12	7.02	7.04	7.10
Class	Rather clean water	Rather clean water	Rather clean water	Rather clean water	Rather clean water	Rather clean water
